# Mitochondrial Sirtuins in Cancer: A Revisited Review from Molecular Mechanisms to Therapeutic Strategies

**DOI:** 10.7150/thno.97320

**Published:** 2024-05-11

**Authors:** Hui Shen, Wei Ma, Yue Hu, Yuan Liu, Yaowen Song, Leilei Fu, Zheng Qin

**Affiliations:** 1 Department of Respiratory and Critical Care Medicine, Department of Breast Surgery, Department of Outpatient, and Department of Radiation Oncology, The First Hospital of China Medical University, Shenyang 110001, China.; 2 Sichuan Engineering Research Center for Biomimetic Synthesis of Natural Drugs, School of Life Science and Engineering, Southwest Jiaotong University, Chengdu 610031, China.

**Keywords:** Mitochondrial sirtuin, Cancer therapy, Small-molecule compound, SIRT3, SIRT4, SIRT5

## Abstract

The sirtuin (SIRT) family is well-known as a group of deacetylase enzymes that rely on nicotinamide adenine dinucleotide (NAD^+^). Among them, mitochondrial SIRTs (SIRT3, SIRT4, and SIRT5) are deacetylases located in mitochondria that regulate the acetylation levels of several key proteins to maintain mitochondrial function and redox homeostasis. Mitochondrial SIRTs are reported to have the Janus role in tumorigenesis, either tumor suppressive or oncogenic functions. Although the multi-faceted roles of mitochondrial SIRTs with tumor-type specificity in tumorigenesis, their critical functions have aroused a rising interest in discovering some small-molecule compounds, including inhibitors and activators for cancer therapy. Herein, we describe the molecular structures of mitochondrial SIRTs, focusing on elucidating their regulatory mechanisms in carcinogenesis, and further discuss the recent advances in developing their targeted small-molecule compounds for cancer therapy. Together, these findings provide a comprehensive understanding of the crucial roles of mitochondrial SIRTs in cancer and potential new therapeutic strategies.

## 1. Introduction

Mitochondrial sirtuins (SIRTs) family proteins SIRT3, SIRT4 and SIRT5 are located in mitochondria and can regulate oxidative phosphorylation of enzymes and other mitochondrial proteins to produce ATP 1. SIRT3 is the most important mitochondrial deacetylase, located in the mitochondrial matrix, and plays an important role in regulating mitochondrial metabolism and oxidative stress, including the tricarboxylic acid cycle, urea cycle, amino acid metabolism, fatty acid oxidation, mitochondrial electron transport chain/oxidative phosphate, reactive oxygen species (ROS) detoxification, mitochondrial dynamics and mitochondrial folding protein response 2. Mitochondrial dysfunction plays a key role in tumor initiation, progression and metabolic reprogramming 3. In recent years, increased anticancer drugs targeting mitochondria have been developed. As a key mitochondrial deacetylase, the role of SIRT3 in tumor has attracted extensive attention. SIRT4 protein has NAD dependent activities of proteolipase, ADP ribosomal transferase and lysine deacylase 4, while SIRT5 protein has NAD dependent activities of lysine deacyloylase, desuccinylase and desglutarylase 5. SIRT4 and SIRT5 are widely involved in biological processes such as cell survival, aging and metabolism 6, 7. They play important roles in many biological processes such as biological stress response, inflammatory response, tumorigenesis and energy metabolism, and exhibit potential therapeutical effects in various diseases such as cancer, diabetes, obesity, Parkinson's disease and Alzheimer's disease 8, 9.

The metabolic pattern of tumor cells is different from that of normal cells. The metabolic reprogramming of tumor cells is characterized by the "Warburg effect", most cancer cells rely more on aerobic glycolysis to provide energy 3. Glycolysis not only provides rapid energy supply, but also provides many favorable factors for the occurrence and development of tumor microenvironment, such as accelerating genomic instability, activating phosphatidylinositol 3 kinase (PI3K)/protein kinase B (Akt), c-myc and other cell proliferation signals. However, OXPHOS is still the main energy supply mode for glioblastoma, neuroblastoma and acute myeloid leukemia 10. Different tumor microenvironments vary greatly, and the survival pressure makes them choose the most appropriate metabolic pathway. As a key regulator of tumor metabolism, SIRT3 participates in the metabolic reprogramming of mitochondria and can regulate the level of ROS to play a role in tumor inhibition, which is a significant feature of SIRT3. However, a recent study found that SIRT3 was transformed into a carcinogenic gene, which can promote the tumorigenesis induced by high-fat diet in mice 11. Therefore, the role of SIRT3 in tumor may be related to genetic background and environment. The expression of SIRT3 was up-regulated during food restriction and caloric restriction. Under stress conditions, SIRT3 knockout cells showed genomic instability and tumorigenesis potential. SIRT3 expression was absent in 40% of breast cancer. In other types of cancer, SIRT3 expression did not show loss but decreased 12. Moreover, most tumor cells must use glutamine efficiently, such as breast cancer cells, cervical cancer cells and liver cancer cells. Without exogenous glutamine supply, they cannot survive 13. The reprogramming of glutamine metabolism in tumor cells is mainly to increase glutamine uptake and accelerate catabolism 14. At present, SIRT4 has been confirmed to inhibit the occurrence and development of tumor by inhibiting glutamine metabolism 15. As a tumor suppressor, SIRT4 inhibits glutamine metabolism in mitochondria of tumor cells. Lung cancer occurred in elderly SIRT4 knockout mice. In small cell lung cancer, leukemia, gastrointestinal tumors and breast cancer, the expression of SIRT4 is reduced 16-20. These researches show that SIRT4 is a tumor suppressor. Interestingly, SIRT4 not only acts as a tumor suppressor, but also promotes tumor progression. Stress induced accumulation in cells such as DNA damage can lead to mutation of oncogenes, which plays an important role in the occurrence and development of tumors. DNA damage causes cell cycle arrest, which is a way for cells to maintain genome stability. Glutamine is the key substance for cells from G1 phase to S phase. Studies have confirmed that the expression of SIRT4 is increased under the condition of DNA damage, which leads to cell cycle arrest by inhibiting glutamine metabolism, thus reducing the accumulation of DNA damage and providing sufficient time for DNA damage repair (DDR) and promoting self-protection 21. Therefore, the reduced expression of SIRT4 in non-tumor cells leads to the accumulation of cell mutations and DNA damage, which contributes to the formation of tumors. In a tissue microarray staining study of 94 cases of breast cancer and adjacent non tumor tissues, the researchers found that the expression level of SIRT4 in breast cancer tissues was significantly higher than that in adjacent tissues, and this study also proved that SIRT4 could promote the proliferation, migration and invasion of breast cancer cells 22. There are few studies on the relationship between SIRT5 and tumor. Recent studies have found that SIRT5 can delay the development of hepatocellular carcinoma (HCC) as a tumor suppressor 23. In addition, the expression level of SIRT5 decreased in endometrial cancer 24, head and neck squamous cell carcinoma [25]and glioma 26. Other studies have shown that SIRT5 can play the role of oncogenes. While the expression of SIRT5 is higher, the prognosis of cancer is worse 27, 28. SIRT5 can promote tumor proliferation, metastasis, drug resistance and metabolic reprogramming through a variety of mechanisms 29, 30.

The regulator of mitochondrial SIRTs plays an important role in treating various diseases and maintaining physiological balance. Exploitation of SIRT3 activators for cancer treatment is still in the early stages. SIRT3 small molecule activators are primarily designed by using several distinct strategies, and the first one involves obtaining small molecule drugs that specifically target SIRT3 by modifying the 1,4-dihydropyridyl structure of the pan-SIRTs activators. Moreover, SIRT3 inhibitors also play an important role for treating cancer, and the SIRT3 inhibitor development methodologies are more varied compared to SIRT3 activators. 3-TYP is a highly potent SIRT3 inhibitor that was originally recognized to have certain anticancer effects in leukemia, non-small cell lung cancer and colorectal cancer, and has been widely used in the researches of various cancer due to its powerful inhibition of deacetylation activity of SIRT3 31. The development of small molecule inhibitors targeting SIRT4 remains considerably constrained, with the majority of investigations yielding inhibitors of low potency and non-selectivity. Compound 69 stands out as the first selective inhibitor with commendable activity against SIRT4 4. Leveraging SIRT5's unique acyl preference, He *et al.* synthesized thiosuccinyl peptides and validated that the H3K9TSu peptide is a selective competitive inhibitor targeting SIRT5, marking the first specific inhibitor for SIRT5 32.

Thus, in this review, we demonstrate the molecular structures and biological functions of mitochondrial SIRTs (SIRT3, SIRT4 and SIRT5), summarize their oncogenic and tumor suppressive roles in carcinogenesis and further discuss a series of targeted small-molecule compounds to improve potential cancer therapy.

## 2. Mitochondrial sirtuins biology

### 2.1 Structure of genes and proteins

**Figure 1:**
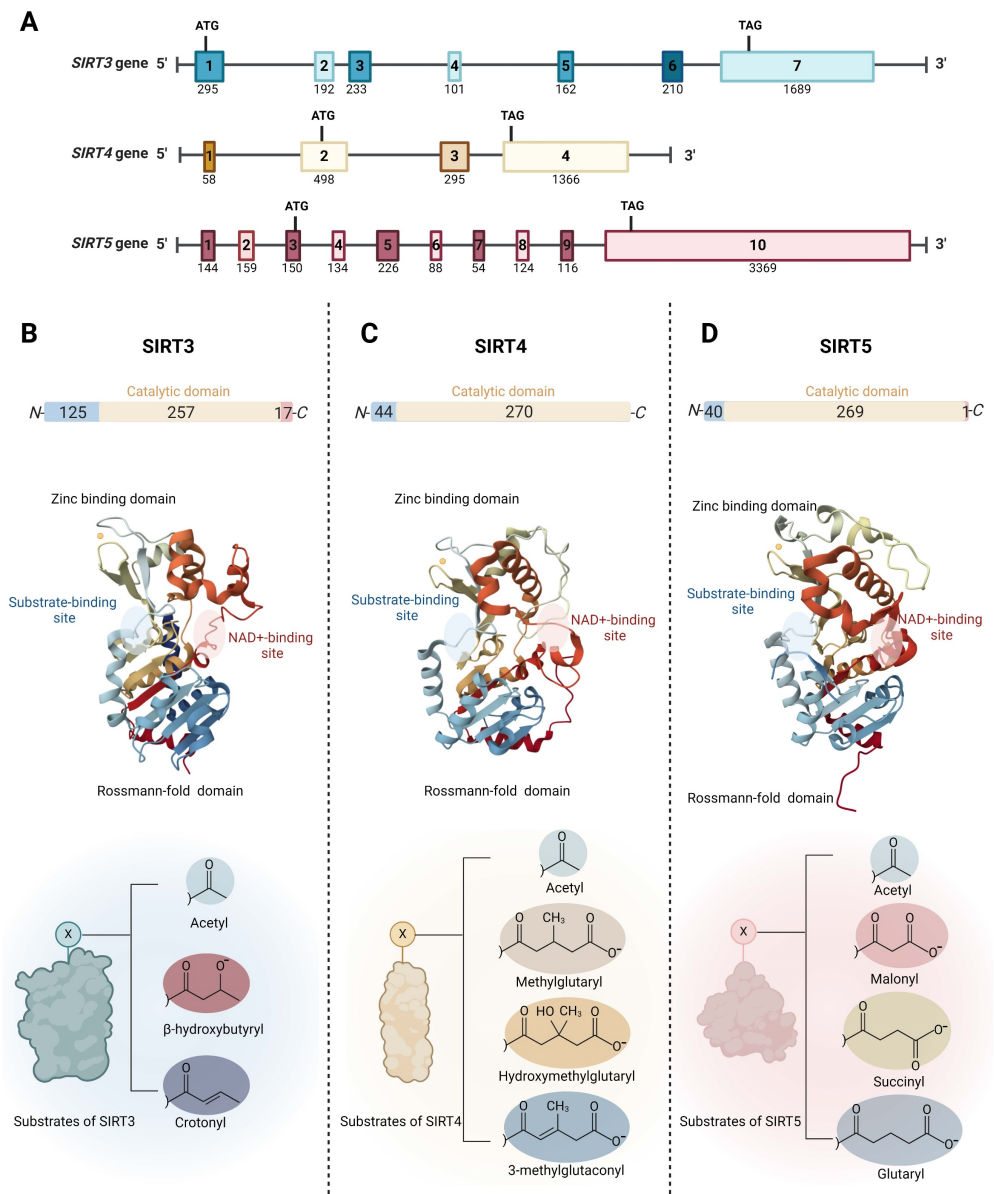
** Genomic and molecular structures of the mitochondrial sirtuins (SIRT3, SIRT4 and SIRT5).** (A) Schematic view of the genomic structure of mitochondrial *SIRT* genes. The boxes mark the exons. Start and stop codons are indicated. The numbers on the genes refer to exons. The numbers below the genes indicate the sizes of the exons. (B) SIRT3 (PDB ID: 3GLS), (C) SIRT4 (PDB ID: 5OJ7), and (D) SIRT5 (PDB ID: 3RIY), are characterized by the presence of two domains, the Rossmann-folding domain and the zinc-binding domain. The different substrate-binding sites of mitochondrial SIRTs allows them to exhibit distinct substrate preferences and catalytic activities.

The *SIRT3* gene is located in the 15.5 region of the short arm of chromosome 11 (11p15.5). It spans approximately 21 kilobases (kb) and consists of seven exons, all of which are coding. The *SIRT4* gene, located on chromosome 12 at position 12q24.31, covers about 11 kb and contains four exons; three are coding and one is non-coding. The *SIRT5* gene is situated in the 23 region of the short arm of chromosome 6 (6p23) and extends over 40 kb. It comprises ten exons, eight of which are coding and two non-coding (Fig. 1A).

Mitochondrial SIRTs share a conserved protein structure characterized by a catalytic core region composed of approximately 275 amino acids, including a large Rossmann-fold domain for NAD binding and a smaller and more variable zinc-binding domain with connecting loops forming extended crevices for substrate entry (Fig. 1B-D) 33, 34. The catalytic core facilitates the deacylation mechanism common to the sirtuin protein family, involving NAD and acetyl-lysine substrate binding, nicotinamide riboside bond cleavage, acetyl transfer, and product formation 35. Specifically, the highly conserved Rossmann-fold domain is notable for its typical NAD-binding features, comprising a central β-sheet formed by six parallel β strands, flanked by multiple α-helices on both sides (the number of α-helices varies among specific sirtuin proteins) 36. The small zinc-binding domain, characterized by its variability in primary sequence, three-dimensional structure, and position, results from insertions in the Rossmann-fold domain and comprises a three-stranded β sheet and a sirtuin-dependent α helical region 37. The cleft region connecting the loops of both the Rossmann-fold domain and the zinc-binding domain functions as the enzymatic active site for the binding of NAD and acetylated lysine substrates. This region is highly conserved in sequence among the sirtuin protein family and plays a crucial role in sirtuin catalysis 38. The variability of the acyl substrate-binding cleft allows mitochondrial SIRTs to exhibit distinct substrate preferences and a range of deacylase activities. SIRT3 is a strong deacetylase but can also remove β-hydroxybutyryl and crotonyl groups (Fig. 1B) 39. SIRT4 processes a substrate-specific deacetylase activity, but it exhibits the catalytic preferences to remove more complex negatively charged acylations such as methylglutaryl, hydroxymethylglutaryl, and 3-methylglutaconyl groups (Fig. 1C) 40, 41. Furthermore, SIRT4 holds the unique distinction of being the sole sirtuin with ADP-ribosyltransferase activity in mitochondria 39. In addition to deacetylation, SIRT5 prefers to catalyze demalonylation, desuccinylation, and deglutarylation of lysine (Fig. 1D) 42, 43.

Outside the conserved catalytic core, mitochondrial SIRTs possess variable N- and C- terminals. Mitochondrial SIRTs possess an N-terminal mitochondrial localization sequence (MLS) of varying lengths and sequences, which can be recognized by the mitochondria, facilitating the entry of SIRT proteins through the mitochondrial membrane into the interior. Specifically, SIRT3's MLS comprises approximately 100 amino acid residues, SIRT4 contains 28 amino acids, and SIRT5 consists of 36 amino acids. Upon mitochondrial entry, SIRT3 is cleaved by the mitochondrial processing peptidase (MPP) at its N-terminus, thus forming its active conformation 33. Similar to SIRT3, SIRT5 has an N-terminal pre-sequence that is cleavable. For SIRT4, the 28 residues at its N-terminus are also reported to undergo post-translational cleavage 44. SIRT3 and SIRT5 have shorter C-terminal extensions compared to other SIRTs, while SIRT4 lacks a C-terminal extension 45. It has been reported that SIRT5 mRNA undergoes alternative splicing, resulting in two variants with different lengths of C-terminal residues, exhibiting distinct intracellular localizations 46, highlighting the importance of the C-terminal extension in influencing the subcellular localization of mitochondrial SIRTs.

### 2.2 Biological functions

SIRT3 plays a critical role in regulating mitochondrial function and cellular stress responses. Primarily located within the mitochondria, SIRT3 is a NAD^+^-dependent deacetylase, which means it relies on nicotinamide adenine dinucleotide to remove acetyl groups from various protein substrates. This activity is essential for the regulation of metabolic processes, including fatty acid oxidation, the tricarboxylic acid (TCA) cycle, and the electron transport chain 33, 47. Functionally, SIRT3 has been shown to deacetylate and activate numerous enzymes critical for mitochondrial metabolism 33. For instance, it activates acetyl-CoA synthetase 2 (ACS2) and the long-chain acyl-CoA dehydrogenase (LCAD), enhancing fatty acid utilization 48. SIRT3 also modulates the activity of complex I of the electron transport chain, thereby influencing ATP production and reducing ROS levels. By deacetylating isocitrate dehydrogenase 2 (IDH2) and superoxide dismutase 2 (SOD2), SIRT3 boosts antioxidant defenses and reduces oxidative damage 47. The role of SIRT3 extends beyond metabolism to include aging and cellular stress responses. Studies have linked SIRT3 to longevity and caloric restriction, suggesting that its enzymatic activity contributes to the healthspan and lifespan extension observed in various models of dietary restriction.

Moreover, SIRT3 has protective roles in cardiac stress, neurodegeneration, and other disease states, making it a potential therapeutic target for metabolic and age-related diseases 33. Through its broad range of substrates and effects, SIRT3 exemplifies how targeted modulation of mitochondrial enzymes can have profound impacts on cellular function and overall health.

Unlike some other SIRTs, SIRT4 lacks robust deacetylase activity but is characterized by its ADP-ribosyltransferase activity 40, 41. One of the key functions of SIRT4 involves the regulation of amino acid metabolism through its inhibition of glutamate dehydrogenase (GDH) 49. This inhibition by SIRT4 can impact amino acid utilization, potentially affecting energy production and insulin secretion, which links it directly to metabolic control and cellular energy balance 50. SIRT4's ability to regulate insulin secretion highlights its role in maintaining glucose homeostasis, which is crucial for preventing metabolic disorders. In addition to its regulatory functions in amino acid metabolism, SIRT4 is involved in the modulation of fatty acid metabolism. It acts on enzymes that are critical in lipid processing, which plays a part in how cells manage fatty acid usage as an energy source 50. Furthermore, SIRT4's involvement in mitophagy regulation and its potential role in apoptosis underscore its importance in mitochondrial health and overall cellular stress responses 51, 52. These functional characteristics of SIRT4 demonstrate its significant impact on cellular metabolism and its potential as a therapeutic target in treating metabolic diseases and conditions associated with mitochondrial dysfunction.

SIRT5 exhibits unique enzymatic activities, including desuccinylation, demalonylation, and deglutarylation 42, 43. These post-translational modifications enable SIRT5 to regulate various metabolic enzymes and pathways, highlighting its crucial role in cellular metabolism. SIRT5's ability to remove succinyl, malonyl, and glutaryl groups from lysine residues on proteins influences several key metabolic processes 42, 43. For instance, it desuccinylates and activates carbamoyl phosphate synthetase 1 (CPS1), a critical enzyme in the urea cycle, which is essential for ammonia detoxification in the liver 53. This modification by SIRT5 enhances CPS1 activity, thereby facilitating the efficient removal of ammonia and preventing ammonia toxicity. Moreover, SIRT5 has been shown to regulate the activity of enzymes involved in the TCA cycle, fatty acid oxidation, and mitochondrial respiration by removing malonyl and glutaryl groups, which can inhibit these enzymes' activities 53. By controlling these modifications, SIRT5 ensures optimal mitochondrial function and energy production, which are vital for maintaining cellular energy homeostasis. The regulation of these metabolic pathways by SIRT5 underscores its potential as a therapeutic target for metabolic diseases and conditions involving altered mitochondrial function or metabolic imbalance.

## 3. Molecular mechanisms of mitochondrial sirtuins in cancer

**Figure 2:**
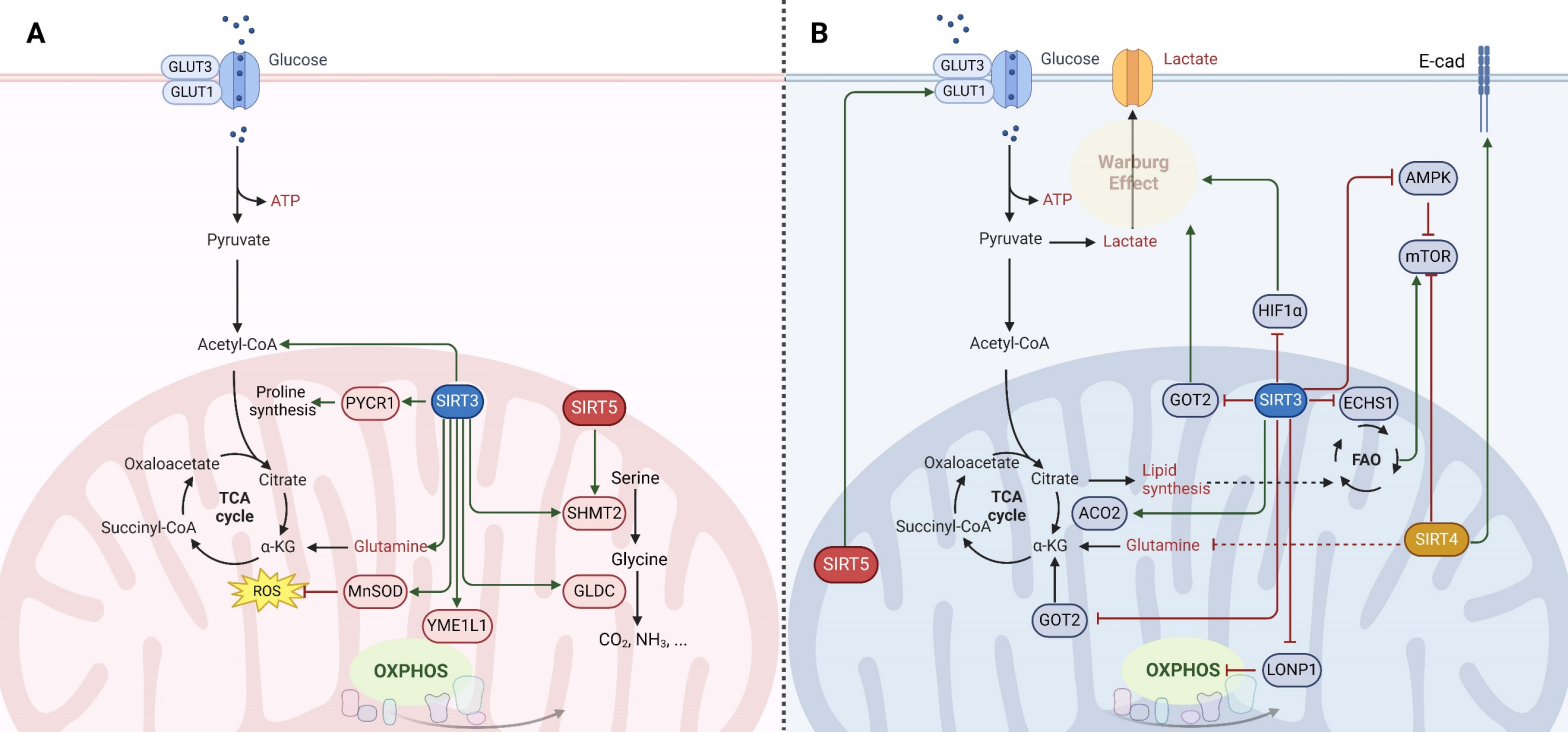
** Molecular mechanisms of mitochondrial sirtuins (SIRT3, SIRT4 and SIRT5) in cancer.** The function of mitochondrial SIRTs in oncogenesis is multifaceted, serving as (A) oncogenes (indicated in red) or (B) tumor suppressors (indicated in blue) contingent upon the genetic background, alongside the tumor type and developmental stage. SIRT3 engages in the deacetylation and activation of various enzymes involving in the tricarboxylic acid (TCA) cycle, fatty acid oxidation (FAO), and oxidative phosphorylation (OXPHOS), thereby manifesting both tumor-promoting and tumor-suppressive capacities under specific circumstances. SIRT4 is endowed with ADP-ribosyltransferase activity and orchestrates the regulation of enzymes critical for amino acid metabolism, lipid metabolism, and the preservation of mitochondrial DNA. The role of SIRT4 in carcinogenesis is primarily viewed as tumor-suppressive; however, similar to SIRT3, effects specific to the context have been documented. SIRT5 modulates the urea cycle and mitochondrial FAO, thereby influencing cellular energy generation and redox equilibrium. This modulation impacts the metabolism and viability of cancer cells, thereby determining its role as either a promoter or suppressor of tumorigenesis.

Mitochondrial SIRTs, through their regulation of mitochondrial metabolism and function, play critical roles in cancer. Their activities influence cancer cell proliferation, survival, and metastasis by modulating metabolic pathways, oxidative stress response, and genomic stability. The dual roles of these SIRTs as both oncogenes (Fig. 2A) and tumor suppressors (Fig. 2B) highlight the intricate relationship between mitochondrial function and cancer, underscoring the potential of targeting mitochondrial SIRTs for cancer therapy. However, the context-dependent nature of their functions necessitates a deeper understanding of the underlying mechanisms to effectively harness their therapeutic potential.

### 3.1 Molecular mechanisms of SIRT3 in cancer

SIRT3, a mitochondrial deacetylase, plays a context-dependent role in cancer as both a tumor suppressor and an oncogene, influenced by the specific circumstances 39. This dualistic effect of SIRT3 is closely associated with the metabolic requirements of cancer cells, which pivot between aerobic glycolysis and oxidative phosphorylation for energy production. The complexity of SIRT3 function is further magnified by its involvement in various regulated cell death (RCD) pathways, where it has been shown to exhibit both tumor-suppressive and tumor-promoting effects. Additionally, the contribution of SIRT3 to enhance mitochondrial function may facilitate epithelial-mesenchymal transition (EMT) and modulate the immune response under specific conditions. A comprehensive understanding of the multifaceted role of SIRT3 is essential to effectively exploit its therapeutic potential in cancer.

SIRT3 has emerged as a pivotal regulator of cancer cell metabolism, oscillating between oncogenic and tumor-suppressive roles. Tumor cells typically exhibit increased glycolysis even under oxygen-rich conditions, a phenomenon known as the Warburg effect 3. SIRT3 acts as a tumor suppressor by undermining the Warburg effect through destabilizing HIF1α, a key transcription factor for glycolytic gene expression 3. SIRT3 activates prolyl hydroxylase (PHD) to hydroxylate hypoxia-inducible factor-1α (HIF1α), reducing its stability and diminishing its pro-tumorigenic activity in glycolytic malignancies 39. Additionally, SIRT3 impedes the malate-aspartate shuttle by deacetylating glutamate oxaloacetate transaminase 2 (GOT2), curtailing glycolysis and thus inhibiting the growth of pancreatic tumor cells 54. SIRT3 also deacetylates and inactivates cyclophilin D, which affects glucose uptake, lactate, pyruvate, and acetyl-coenzyme production, suppressing glycolysis in colon carcinoma 55. Moreover, SIRT3 enhances mitochondrial functions and induces metabolic reprogramming in clear cell renal cell carcinoma, which leads to reduced glucose uptake and increased sensitivity to anticancer drugs (e.g., resveratrol, everolimus, and temsirolimus), highlighting its potential role in improving therapeutic outcomes 56. Additionally, SIRT3 also reprograms cellular lipid metabolism in tumor cells by regulating lipid metabolism enzymes. For instance, SIRT3 can counteract the oncogenic acetylation of enoyl-coenzyme A (CoA) hydratase-1 (ECHS1), reducing the accumulation of fatty acids and branched-chain amino acids that activate oncogenic mammalian target of rapamycin (mTOR) signaling 57. Furthermore, the suppression of SIRT3 expression has been shown to enhance mitochondrial aconitase 2 (ACO2) activity, thereby facilitating mitochondrial citrate synthesis and contributing to the progression of prostate cancer 58. Conversely, in certain cancers where oxidative phosphorylation (OXPHOS) remains the primary energy source, SIRT3 can function as an oncogene 39. SIRT3 contributes to cancer progression by deacetylating subunits of the electron transport chain (ETC) and enhancing OXPHOS, thus promoting mitochondrial biogenesis and function 39, 59. In chronic lymphocytic leukemia cells, upregulation of SIRT3 activates the manganese superoxide dismutase (MnSOD) to counteract the harmful effects of excessive superoxide, thus favoring cell survival 60. Besides, SIRT3 facilitates the survival and function of leukemia stem cells in acute myeloid leukemia by regulating fatty acid oxidation essential for OXPHOS and ATP production in leukemia stem cells 61. Similarly, SIRT3 in glioma stem cells enhances mitochondrial function and confers metabolic flexibility, thereby maintaining stemness, resistance to stress, and promoting tumor formation in glioblastoma 62. Nonetheless, SIRT3 deacetylates the oncogene Lon protease-1 (LONP1) to reduce the energy supply of OXPHOS, which restrains primary tumor growth, highlighting its context-dependent roles in carcinogenesis 63.

Beyond energy metabolism, SIRT3 also exerts a significant influence on amino acid metabolism, thereby ensuring an adequate supply of amino acids for cancer cells and promoting tumorigenesis. For instance, SIRT3 deacetylates and activates pyrroline-5-carboxylate reductase 1 (PYCR1), catalyzing proline synthesis and promoting cell proliferation 64. Similarly, the deacetylation and activation of serine hydroxymethyltransferase 2 (SHMT2) by SIRT3 enhances the conversion of serine to glycine, promoting colorectal cancer cell proliferation by increasing serine consumption and NADPH levels 65. Moreover, SIRT3 induces deacetylation of glycine decarboxylase (GLDC) and promotes glycine catabolism and pyrimidine synthesis, which is crucial for the proliferation of human glioma cells 66. Thus, SIRT3's influence on cancer appears to be context-dependent, acting as either an oncogene or a tumor suppressor based on the specific metabolic demands of the cancer type. This dualistic nature of SIRT3 underscores the intricacies of cancer metabolism and highlights the nuanced interplay between metabolic pathways and tumor progression.

SIRT3 is also involved in various RCD pathways to exhibit distinct functions, depending on the cellular context and cancer type. In terms of its tumor-suppressive functions, SIRT3 induces apoptosis by activating key components of the apoptotic machinery. For instance, SIRT3 activates glycogen synthase kinase-3β (GSK-3β), which induces the expression and mitochondrial translocation of Bax to promote apoptosis, thereby inhibiting cell growth both *in vitro* and *in vivo* 33. Additionally, SIRT3 upregulates the activities of p53, further enhancing apoptosis and inhibiting pancreatic cancer cell proliferation 67. SIRT3 also deacetylates mutant p53, inducing apoptosis in small-cell lung cancer and enhancing chemosensitivity to cisplatin 68. SIRT3-mediated delactylation of cyclin E2 (CCNE2) curbs hepatocellular carcinoma cell growth by inducing apoptosis 69. Intriguingly, the role of SIRT3 in autophagy is context-dependent, with evidence suggesting it can both inhibit tumor progression and protect tumor cells under stress. For example, SIRT3 can prevent autophagy by enhancing glutamine flux to the TCA cycle and maintaining acetyl-CoA pools, thus supporting the proliferation, survival, and self-renewal of diffuse large B cell lymphomas 70. Conversely, in breast cancer, SIRT3-mediated autophagy via the 5'-adenosine monophosphate-activated protein kinase (AMPK)-related pathway suppresses cell proliferation, migration, and invasion 71. SIRT3 promotes the doxorubicin (DOX)-resistance of lung cancer cells by enhancing autophagy to counteract DOX-induced senescence primarily through the inhibition of PI3K/AKT/mTOR signaling 72. SIRT3 enhances DNA damage repair and promotes radiation resistance in colorectal cancer cells by inducing mitophagy 73. Furthermore, SIRT3's involvement in ferroptosis adds another layer of complexity to its role in cancer. SIRT3 can inhibit AKT-dependent mitochondrial metabolism in gallbladder cancer, thereby inducing ferroptosis and tumor suppression 74. SIRT3 knockdown leads to the downregulation of SLC7A11, consequently sensitizing glioblastoma to ferroptosis both *in vitro* and *in vivo* by disrupting iron metabolism and promoting mitophagy 75.

Additionally, SIRT3 has been reported to play a dual role in cancer by affecting EMT and tumor immune response. SIRT3 hinders EMT by activating AMPK and inhibiting mTOR/HIF-1α pathway, which further inhibits cancer-associated fibroblast-mediated tumorigenesis in non-small cell lung cancer (NSCLC) 76. Moreover, SIRT3 activation promotes stemness, metastasis, and EMT in NSCLC by activating the Wnt/β-catenin pathway through modulating the deacetylation of replication timing regulatory factor 1 (RIF1) 77. Conversely, SIRT3 can enhance the cell proliferation, invasion, and EMT of triple-negative breast cancer cells by promoting mitochondrial biogenesis and function via MnSOD activation 78. Monitoring mitochondrial metabolites like lactate in the tumor microenvironment may offer insights into modulating SIRT3 for improved cancer treatment outcomes. While SIRT3 deacetylates mitochondrial metalloprotease YME1L1 to promote mitochondrial fusion and OXPHOS, thereby facilitating T cell infiltration into tumor tissue and enhancing anti-tumor immunity 79. Conversely, in prostate cancer, SIRT3 suppresses the recruitment of macrophages and neutrophils to dampen the innate immune response and contribute to cancer progression 80.

The complex role of SIRT3 in cancer underscores the nuanced challenge of developing effective SIRT3 agonists and inhibitors as therapeutic agents. SIRT3's dualistic nature, acting as both a tumor suppressor and an oncogene depending on the cancer context, highlights the critical importance of targeted therapy. For example, in cancers where SIRT3 suppresses tumor growth by undermining the Warburg effect or inducing apoptosis, SIRT3 agonists could be beneficial. Conversely, in scenarios where SIRT3 supports cancer progression by enhancing oxidative phosphorylation or promoting cell survival mechanisms, SIRT3 inhibitors might be more appropriate. The challenge in drug development lies in the specificity and timing of SIRT3 modulation. Therapeutic strategies need to carefully consider the cancer type, stage, and the specific metabolic pathways involved to effectively utilize SIRT3 modulators. Moreover, understanding the regulatory mechanisms of SIRT3 in different cancers can aid in designing drugs that either enhance its tumor-suppressive functions or inhibit its oncogenic activities. In summary, the precision in targeting, based on thorough molecular and cellular profiling of tumors, is essential to harness the potential of SIRT3 as a therapeutic target in cancer treatment.

### 3.2 Molecular mechanisms of SIRT4 in cancer

SIRT4 is characterized as a mitochondrial lipoamidase, demonstrating robust enzymatic activity toward lipoyl- and biotinyl-lysine modifications, particularly with pyruvate dehydrogenase (PDH) as a biological substrate. It is involved in regulating overall PDH activity by hydrolyzing the lipoamide cofactors from dihydrolipoyllysine acetyltransferase (DLAT), thus inhibiting the conversion of pyruvate to acetyl CoA. SIRT4's unique catalytic efficiency and substrate specificity for lipoylated DLAT suggest its pivotal role in regulating PDH activity and cellular metabolism. The temporal regulation of SIRT4 lipoamidase activity, induced by glutamine stimulation, further underscores its significance in PDH inhibition and cellular metabolic responses 40, 81. ADP-ribosyl transferase activity is showcased by SIRT4, modulating glutamate dehydrogenase and diminishing insulin secretion in β cells 82. Recent studies have demonstrated that SIRT4 inhibits tumor growth in pancreatic ductal adenocarcinoma through in vitro and in vivo experiments. The mechanism involves SIRT4-mediated suppression of glutamine metabolism, leading to activation of p53 phosphorylation and subsequent autophagy induction. Numerous studies have also shown the important role of SIRT4 in various tumors 83.

SIRT4 downregulation in colorectal cancer (CRC) suggests its tumor-suppressive role. Previous research demonstrated SIRT4's inhibition of tumor growth by targeting glutamine metabolism. Knockout of SIRT4 in CRC cells increased proliferation, migration, and invasion, confirming its tumor-suppressive effects. Moreover, SIRT4 knockout reduced the sensitivity of CRC cells to chemotherapy drugs like 5-FU and oxaliplatin. Mechanistically, SIRT4 knockout inhibited apoptosis in response to 5-FU, implicating its role in chemosensitivity. These findings suggest SIRT4 as a potential therapeutic target in CRC, offering new insights into its modulation of tumor biology and chemotherapy response 84. Additionally, SIRT4 overexpression impedes proliferation, migration, and invasion of CRC cells, suggesting its therapeutic potential. Notably, SIRT4 downregulation in CRC tissues correlates with adverse outcomes. Mechanistically, the cell cycle is arrested by SIRT4 through regulation of cyclins and ERK, and apoptosis-related proteins like caspases and NF-κB are modulated 15. Tumor-suppressive functions in CRC are exerted by SIRT4 through regulation of E-cadherin expression and inhibition of EMT via suppression of glutamine metabolism. It positively influences E-cadherin expression, crucial for maintaining epithelial characteristics and inhibiting cancer cell invasion and metastasis. Worse prognosis and advanced CRC stages correlate with low SIRT4 levels, indicating its potential as a prognostic marker. Thus, targeting SIRT4 and its regulatory pathways in CRC could offer promising therapeutic avenues for treating colorectal tumors 85. Localized within mitochondria, SIRT4 maintains mitochondrial integrity and influences cellular metabolism, oxidative stress response, and potentially autophagy, although further research is needed to elucidate its precise role in this process 86, 87. In addition, research proves the significant role of SIRT4 in modulating mitochondrial glutamine metabolism and proliferation in Myc-driven tumor models of breast cancer. Remarkably, Myc-induced lymphomagenesis in mice is accelerated by SIRT4 loss, highlighting its tumor-suppressive function. SIRT4 emerges as a tumor suppressor in various cancers, evidenced by multiple studies. It inhibits tumor growth by suppressing glutamine metabolism, as observed in HeLa cells and human colon and prostate cancer cell lines. Promotion of tumor formation in MEF cells and spontaneous tumor development in mice, including lung, liver, breast, and lymphomas, is seen with SIRT4 deficiency. Moreover, tumor progression is delayed in xenograft models with SIRT4 overexpression. Analysis of expression databases reveals SIRT4 downregulation in several human cancers, including breast, bladder, gastric, colon, thyroid, and ovarian cancers. In gastric adenocarcinoma, low SIRT4 expression correlates with increased malignancy, affirming its tumor suppressive role 88.

The importance of mitochondrial glutamine metabolism in oncogene-induced tumorigenesis is underscored by these findings, and SIRT4 is highlighted as a critical regulator in this process. The regulation of SIRT4 expression in cancer is complex, involving factors such as mTORC1-mediated degradation of CREB2, which controls SIRT4 transcription. It was revealed that mTORC1 enhances glutamine metabolism by upregulating Glutamate dehydrogenase (GDH) activity via the repression of SIRT4 expression, mediated by proteasome-mediated degradation of the SIRT4 transcriptional regulator CREB2 89. This newly identified mechanism contributes to the repertoire of transcriptional regulators modulated by the mTORC1 pathway, alongside HIF1α, Myc and SREBP1. The findings suggest a cooperative regulation between CREB2/SIRT4 and Myc/GLS axes, crucial for controlling glutamine metabolism and α-ketoglutarate (α-KG) flux. Reduced SIRT4 expression in human cancers correlates with increased cell transformation and tumor development, highlighting its role in tumor metabolism. Additionally, the study underscores the therapeutic potential of targeting glutamine metabolism in cancer treatment, synergizing with glycolytic attenuation to induce tumor cell death 90. Overall, SIRT4 exerts dual functions in cancer by modulating glutamine metabolism, suggesting its potential as a therapeutic target for cancer treatment, although further studies are needed to elucidate its precise mechanisms and therapeutic implications in different cancer contexts. Notably, SIRT4's function extends beyond metabolic regulation, implicating its involvement in diverse cellular processes, including insulin secretion, fatty acid oxidation, and tumor suppression. The identification of SIRT4 as a crucial regulator of mitochondrial function and cellular metabolism offers new insights into the intricate mechanisms underlying metabolic homeostasis and disease pathogenesis, warranting further investigation into its implications in various health conditions.

### 3.3 Molecular mechanisms of SIRT5 in cancer

SIRT5 is primarily involved in regulating protein post-translational modifications such as lysine succinylation, malonylation, and glutarylation. Desuccinylation, demalonylation, and deglutarylation activities are exhibited by SIRT5, which distinguishes it from other SIRTs. It represses isocitrate dehydrogenase 2 (IDH2) activity and may disrupt glutamine metabolism through glutaminase (GLS), thus impacting cellular respiration and fatty acid oxidation. SIRT5 also plays a role in modulating glutamine metabolism, potentially influencing cancer progression. Moreover, it contributes to cellular oxidative stress regulation by targeting downstream factors such as HIF-1α and eNOS, and by modulating ROS formation. Sirt5-deficient cells unveils elevated ROS levels, attributed to impaired ROS scavenging rather than increased production. Antioxidant capacity is maintained by SIRT5 through modulation of NADPH-producing enzymes, IDH2, and G6PD. Specifically, K413 on IDH2 is targeted by SIRT5 for succinylation, implicating it in redox regulation 91. Within mitochondria, SIRT5 helps maintain mitochondrial function and homeostasis, with its precise role in processes like mitophagy necessitating further investigation. Recent studies have shown that the expression of SIRT5 negatively impacts tumor cell proliferation in PDAC patients and is associated with a favorable prognosis 92. Additionally, loss of SIRT5 promotes the development of liver cancer by altering bile acid metabolism and fostering an immune-suppressive tumor environment. Targeting this pathway with existing drugs such as cholestyramine may be beneficial for treating HCC patients. These findings collectively highlight the critical role of SIRTs in tumor development and therapy 93.

In CRC, it promotes cell survival by enhancing autophagy via LDHB deacetylation and lysosome acidification. SIRT5 modulates glutamine metabolism at the post-translational level in CRC, wherein SIRT5 silencing disrupted α-KG formation from glutamate, consequently hindering the entry of glutamine-derived metabolites into the TCA cycle, thereby impeding anabolic biosynthesis. The relationship between SIRT5 and GLUD1 in cancer metabolism, particularly in CRC, is of significant interest. GLUD1, known for its role in catalyzing the reversible deamination of glutamate to produce α-KG, plays a pivotal role in α-KG synthesis in cancer cells. The SIRT5-GLUD1 axis not only regulates α-KG synthesis in cancer but also contributes to redox homeostasis and NADPH production essential for cancer cell proliferation 94, 95. This tumor-specific metabolic vulnerability underscores the potential therapeutic application of targeting SIRT5 in CRC. Moreover, it facilitates one-carbon metabolism through SHMT2 desuccinylation, supporting cell survival under glycine and serine deprivation. SIRT5 expression serves as a prognostic biomarker, impacting CRC chemotherapy response to FOLFOX and cetuximab. It also mediates resistance to FOLFOX and cetuximab via succinate accumulation and FOXO3a-induced apoptosis inhibition. SIRT5 modulation reduces CRC cell viability, especially in p53-expressing cells, suggesting its therapeutic potential 96. SIRT5 orchestrates crucial metabolic and DNA integrity processes. SIRT5 depletion reduces intracellular nucleotide levels, inducing DNA damage and apoptosis. Notably, SIRT5 regulates the non-oxidative branch of the pentose phosphate pathway (PPP), pivotal for ribose-5-phosphate (R5P) production and nucleotide synthesis. Mechanistically, transketolase (TKT) is activated by SIRT5 via lysine 281 demalonylation, enhancing R5P generation and nucleic acid synthesis. This activation promotes CRC progression in vitro and in vivo. The interaction between SIRT5 and TKT underscores lysine demalonylation's role in PPP regulation. Overall, these findings illuminate SIRT5's significance in CRC metabolism and DNA integrity, suggesting its potential as a therapeutic target 97.

SIRT5, overexpressed in breast cancer, correlates with poor patient outcomes. Genetic or pharmacological disruption of SIRT5 impedes breast cancer cell growth both in vitro and in vivo, suggesting its potential as a therapeutic target. SIRT5 is primarily targeted towards succinyl-lysine residues, impacting key metabolic pathways like the TCA cycle and amino acid degradation crucial for cancer cell proliferation. Heightened dependency on SIRT5 is exhibited by breast cancer cells, possibly due to increased oxidative stress. Targeting SIRT5 with inhibitors significantly suppresses tumor growth in mouse models without notable toxicity in normal cells. These findings underscore SIRT5's dispensability in normal conditions but essential role in cancer, highlighting its promise as a therapeutic target 98, 99.

## 4. Targeting mitochondrial sirtuins with small-molecule compounds for cancer therapy

### 4.1 Targeting SIRT3 with small-molecule compounds

#### 4.1.1 Small-molecule activators

SIRT3 has been shown to play a key role in a variety of cancers by maintaining mitochondrial homeostasis and participating in multiple metabolic pathways such as oxidative stress, lipid metabolism, and energy metabolism, so targeting SIRT3 could be a beneficial treatment strategy for multiple disease including heart failure and cancer 100. Due of SIRT3's context-dependent role in different types of cancer, a variety of SIRT3 activators and inhibitors have been developed to regulate cancer growth. The clarification of the crystal structure of SIRT3 has expedited the development of SIRT3 small molecular activators and inhibitors, as indicated by the use of SIRT3 structural analysis, which can predict and identify multiple interaction sites (Table 1).

Unlike SIRT3 inhibitors, SIRT3 activators for cancer treatment are still in the early stages of development. SIRT3 small molecule activators are primarily designed using two distinct techniques, and the first one involves obtaining small molecule drugs that specifically target SIRT3 by modifying the 1,4-dihydropyridyl structure of the pan-SIRTs activators. This 1,4-dihydropyridinyl molecule has been found to not interact with the conventional SIRT3 NAD^+^ or acetylated structural pocket instead of binding to the catalytic active site of SIRT3 directly, triggering the deacetylation of SIRT3 on the substrate. SIRT3 can be specifically activated by adding a phenyl, 2-thiazolyl, or 3-quinolinyl ring to C4 of this particular molecular structure. Several SIRT3 activators have been created using a 1,4-dihydropyridine structure, including compound 31, compound 3c and 3d. These small molecule activators can potentially inhibit tumor growth and metastasis by controlling programmed cell death like apoptosis and autophagy-dependent cell death, electron transport pathways, and redox equilibrium 101. Compound 3C, a new chemical structure based on 1,4-dihydropyridine, specifically triggers SIRT3 and enhances its deacetylation function by almost fourfold. Compound 3D enhances the activity of glutamate dehydrogenase and reduces the deacetylation level of MnSOD by activating SIRT3 in thyroid cancer CAL-62 and triple-negative breast cancer MDA-MB-231 cells. Furthermore, compound 3D demonstrates significant anticancer effects through suppressing the proliferation and migration of cancer cells by reducing the expression of HIF-1α, endothelial PAS domain protein 1 (EPAS-1) and carbonic anhydrase IX (CA-IX), and the related proteins involved in epithelial mesenchymal transition 102. Another SIRT3 activators developed by using a structure-based approach to small molecule drug design is compound 33c, which was derived through high-throughput screening and structural optimization of the initial compound amiodarone. Compound 33c can promote autophagy and mitochondrial autophagy by specifically activating SIRT3 through particularly binding to the U pocket next to the NAD^+^ domain of SIRT3. Furthermore, compound 33c prevented the growth and movement of triple-negative breast cancer MDA-MB-231 cells in vivo and in vitro 103. This study offers insights for creating SIRT3 agonists to treat triple-negative breast cancer and suggests potential for treating other types of cancer as well.

Natural compounds, along with synthetic compounds, are valuable resources for identifying small molecule medications. Honokiol, an extracted from plants bisphenol a molecule, has been shown to induce apoptosis and restrict the formation of HCC cells in living organisms in by activating SIRT3. Honokiol regulates the expression level of cyclin-dependent kinase Cyclin E2 (CCNE2) via stimulating the deacylation activity of SIRT3, which leads to the inhibition of the progression of hepatocellular carcinoma (HCC) 69. This also offers clues for the future development of agonists targeting various deacylation activities of SIRT3.

Moreover, the activation of SIRT3 can be enhanced by certain compounds that serve as positive regulators through upregulating its expression. For example, resveratrol can trigger autophagy by upregulating SIRT3 expression and phosphorylating AMPK, which leads to the suppression of breast cancer development and spread 69. Another compound melatonin, an endogenous chemical, increases SIRT3 expression in lung cancer cells and stimulates the pyruvate dehydrogenase complex (PDH) increasing ATP generation. Moreover, Melatonin can reverse the Warburg effect in cancer cells by promoting a shift from aerobic glycolysis to oxidative phosphorylation, which can decrease lung cancer development by elevating SIRT3 levels 69.

In conclusion, there are few approaches available for creating and identifying SIRT3 agonists, with the majority of research on SIRT3 agonists concentrating on breast cancer. Hence, it is essential to assess the effectiveness of the created chemicals in different types of cancers. Furthermore, innovative development tactics are required to design additional SIRT3 small molecule agonists for cancer therapy.

#### 4.1.2 Small-molecule inhibitors of SIRT3

SIRT3 functions as a cancer promoting factor in various types of cancer including pancreatic cancer, cholangiocarcinoma, liver cancer, gallbladder cancer, and prostate cancer. Thus, SIRT3 inhibitors are crucial for treating cancer due to their association with a poor prognosis, and SIRT3 inhibitors development methodologies are more varied compared to SIRT3 activators, including classic structure-based inhibitor design, chemical library-based inhibitor screening, substrate-based competitive inhibitor design, and coenzyme competitive inhibitor design 33. Other targeted small molecular inhibition designs, like covalent inhibitors, proteolysis targeting chimeras (PROTACs), autophagy-targeting chimeras (AUTACs), and autophagosome-tethering compounds (ATTECs), may offer insights for developing a SIRT3 inhibitor 3.

3-TYP is a highly potent SIRT3 inhibitor that was originally recognized to have certain anticancer effects in leukemia, non-small cell lung cancer and colorectal cancer, and has been widely used in the researches of various cancer due to its powerful inhibition of deacetylation activity of SIRT3 31. Despite 3-TYP's high SIRT3 selectivity, its effectiveness in treating malignancies is restricted to initial investigation. Noteworthily, the specific mechanisms by which it inhibits SIRT3 anticancer action have not been investigated, and its full clinical potential remains unexplored.

Substrate-competitive inhibitors like compounds YC8-02, JH-T4, and Biotin-TM3 are developed by targeting the mitochondrial partial phenylmethylcarbamyl group of SIRT2 inhibitors which can inhibit the deacetylation reaction through binding to the NAD domain of SIRT3. YC8-02 inhibits the TCA cycle in Diffuse large B cell lymphomas (DLBCL) by preventing the decrease of glutamate dehydrogenase and acetyl-CoA through SIRT3, leading to autophagic cell death and suppression of DLBCL cell proliferation 70. Unfortunately, the lower specificity of these three compounds results in their susceptibility to off-target SIRT3, thus necessitating further structural modification. An improved SIRT3 inhibitor compound 8 with increased selectivity to SIRT3 was developed from cambinol, a non-selective inhibitor of the SIRTs family, by enhancing hydrophobic aryl substitution at naphthalene ring C6. Compound 8 has been found to exhibit cytotoxic effects in Burkitt lymphoma, diffuse large B-cell lymphoma, colon cancer (HCT116), breast cancer (MCF-7), and non-small cell lung cancer, showing promise for cancer treatment 104. Like the above three compounds, compound P6 can also exert anticancer effects by binding to the NAD site of SIRT3, inhibiting the activity of SIRT3. The quinoline and 2-fluorophenyl components of compound P6 interact with Phe61 and Phe175 to fill these hydrophobic pockets. Demonstrated specific inhibition of SIRT3 105.

Aside from NAD binding sites, SIRT3 inhibitors can also attach to acetylated lysine binding sites to block SIRT3 activity. A study detailed the creation of a SIRT3 selective inhibitor, Compound 8, by high-throughput label-free screening mass spectrometry, can attach to the binding acetylated lysine site and interact with less conserved amino acids. This interaction results in effective inhibition of SIRT3 and strong selectivity towards SIRT3 106. However, the application of compound 8 for cancer therapy requires a further investigation of the effect and specific mechanism of compound 8 on cancer after inhibiting SIRT3. Moreover, analogs of acyl-CoA synthetase short chain family member 2 (ACS2) peptide substrates can be utilized for creating competitive inhibitors of SIRT3 substrates, including 4'-Bromo-resveratrol (4'-BR), a compound from structurally altered version of the natural compound resveratrol. 4'-BR has demonstrated anti-tumor effects in melanoma via triggering apoptosis and cell cycle arrest. Recent investigations have found that 4'-BR can simultaneously inhibit SIRT1 and SIRT3, leading to the regulation of mitochondrial metabolic rearrangement and the inhibition of melanoma cell growth and migration 107. Although 4'-BR is not specific to SIRT3, it also has potential as an antitumor drug due to its strong anticancer activity. This indicates that effective inhibitors capable of targeting other SIRTs families may have the potential to be developed into anti-tumor medications.

Several compounds have been identified that can compete with acetyl substrates for binding sites and function as competitive inhibitors of NAD+ coenzyme. These compounds use 8-mercapto-3,7-dihydro1h-purine-2,6-dione as scaffolds. For example, compound 15 binds to SIRT3 through hydrophobic contact and forms a hydrogen bond with Arg158 side chain guanidine. The phenyl group of Compound 15 is positioned in the hydrophobic pockets at both ends of SIRT3. Compound 15 can operate as a competitive inhibitor against the acetylation peptide of SIRT3 by binding to the acetylation substrate site and inhibiting SIRT3 action 108.

Natural compounds from plants can function as SIRT3 inhibitors, similar to SIRT3 activators. Kaempferol Apigenin has been evaluated as a possible SIRT3 inhibitor by using different computer simulation techniques such as molecular docking, ADMET, and molecular dynamics simulation. It has been observed that it can hinder the deacetylation activity of SIRT3, hence impeding the growth and spread of triple-negative breast cancer 109. Moreover, the expression level of SIRT3 can also be reduced by some compounds, thereby demonstrating the same effect as SIRT3 inhibitors, ultimately exerting antitumor activity and potentially offering therapeutic benefits for cancer treatment. Cisplatin can decrease SIRT3 expression, leading to increased acetylation of Enzymemethylenetetrahydrofolate dehydrogenase 2 (MTHFD2), an enzyme involved in folate metabolism, resulting in decreased NADPH levels in cells. This disrupts the redox equilibrium in the cell and hinders cancer growth 110.

Among the SIRT3 activators and inhibitors introduced above, no compounds targeting SIRT3 for cancer therapy have entered clinical trials, although honokiol has been used as a therapeutic agent for non-small cell lung cancer in China, but the relationship between honokiol and its activation of SIRT3 is ambiguous, and needs to be further explored 111. There are many challenges in targeting SIRT3 for cancer treatment, such as whether these compounds can enter clinical trials, and further in vitro and in vivo evaluations of their antitumor activity and toxicities are needed.

**Figure 3:**
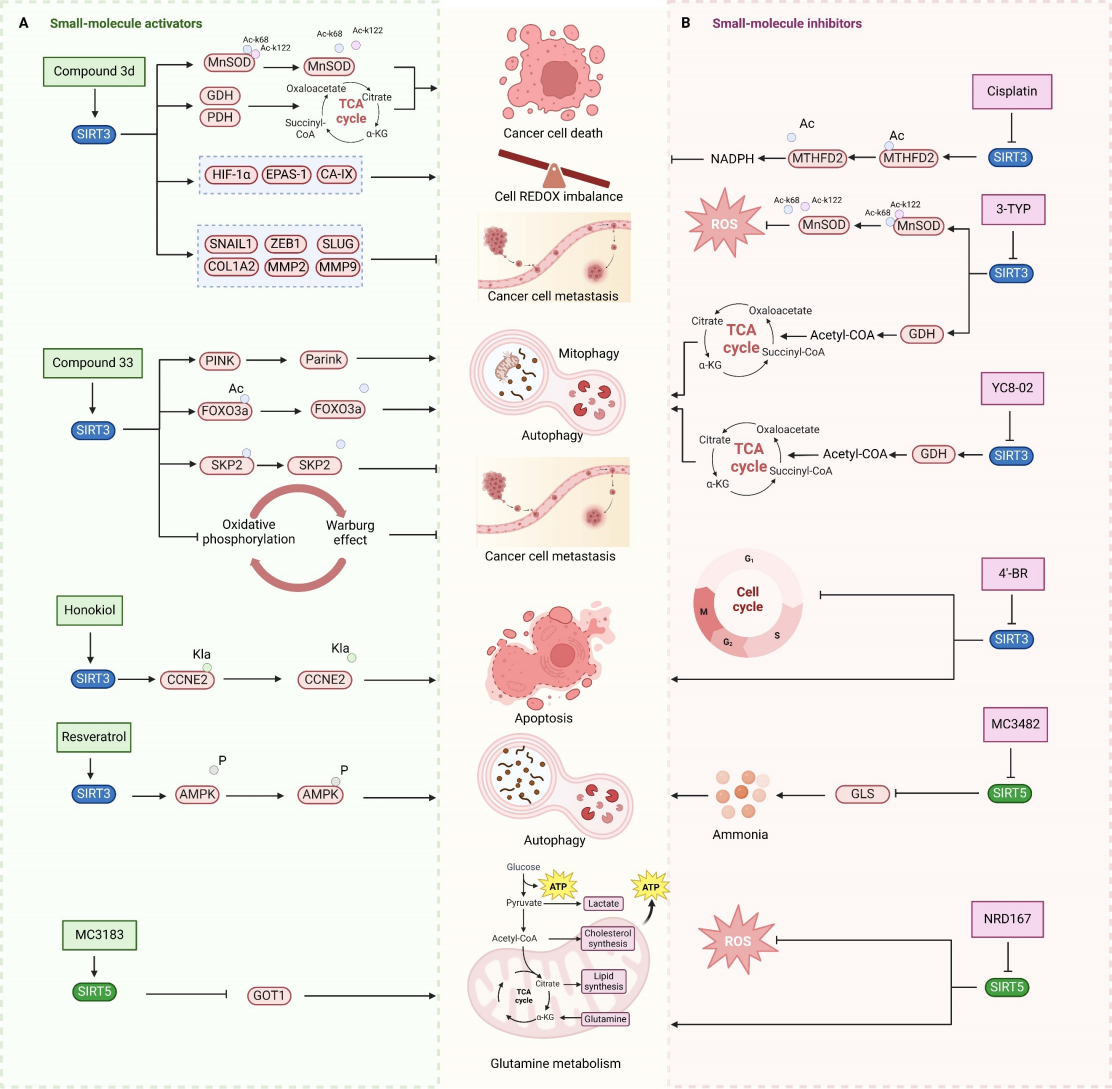
** Small-molecule modulators targeting SIRT3 and SIRT5 for cancer therapy.** SIRT3 and SIRT5 play dual roles in cancer. (A) Small-molecule activators of SIRT3 and SIRT5 and their mechanisms in cancer. (B) Small-molecule inhibitors targeting SIRT3 and SIRT5, as well as their mechanisms in cancer. These modulators regulate tumor proliferation and metastasis by modulating glutamine metabolism, mitochondrial oxidative stress, autophagy, or apoptosis-related pathways. SIRT3 and SIRT5 proteins are represented in blue and green, respectively, and the signaling pathways regulated by small-molecule modulators are indicated in pink.

Overall, despite several efforts to producing SIRT3 inhibitors, there is a shortage of small molecular compounds suitable for cancer treatment by targeting the inhibitor, which indicating that there are numerous problems in developing the SIRT3 inhibitor. Furthermore, due to the crucial involvement of SIRT3 in various physiological processes such as energy metabolism, lipid metabolism, and oxidation restoration, it is essential to take into account the mechanisms by which small molecular compounds target tumors to prevent negative effects on normal cells when developing a selective inhibitor. Finally, many SIRT3 inhibitors have not been tested for their anticancer effects. Thus, evaluating their pharmacological activity based on various roles of SIRT3 in tumors can help identify the most suitable type of tumor for treatment, offering potential for developing new cancer treatment drugs (Fig.3). Furthermore, in addition to the classical SIRT3 small molecule compounds development approaches, a variety of new methods have emerged to be applied to the development of targeted small molecules, such as Sequence-based drug design, Fragment-based drug design and Encoded compound library 112. In order to accelerate the targeting of SIRT3 for cancer therapy, it is necessary to apply these emerging drug design methods to the development of SIRT3 small molecule activators and inhibitors. Furthermore, the modulation of SIRT3 function can also be achieved by targeting its interacting protein network, which has emerged as a potential tumor intervention option by promoting or inhibiting protein-protein interactions.

### 4.2 Targeting SIRT4 and SIRT5 with small-molecule compounds

Diverging from SIRT3, both SIRT4 and SIRT5 demonstrate comparatively diminished deacetylation activity. With the progressive elucidation of the biological functions of SIRT4, multiple regulatory mechanisms in cancer have been unveiled, thus positioning SIRT4 as a prospective therapeutic target for various malignancies **(Table 2).** Nevertheless, the development of small molecule inhibitors targeting SIRT4 remains considerably constrained, with the majority of investigations yielding inhibitors of low potency and non-selectivity. Compound 69 stands out as the first selective inhibitor with commendable activity against SIRT4 4. Pannek *et al.* leveraged a virtual screen based on the crystal structure of SIRT4, derivative design and synthesis premised upon hit compound structures, alongside structure-activity relationship analysis and structural optimization, yielding small molecule inhibitors with good inhibitory efficacy against SIRT4 and heightened subtype selectivity 4. Among these, compounds 60 and 69 respectively exhibit superior potency and maximal selectivity, serving as excellent chemical probes for interrogating the biological functions of SIRT4. Furthermore, compound 69, as the first lead compound for selective inhibition of SIRT4, holds promise in furnishing insights for the further development of SIRT4-targeting modulators. Notably, SIRT4 plays a pivotal role in the suppression of tumorigenesis across a spectrum of cancer types (such as colorectal cancer, breast cancer, prostate cancer, etc.), consistent with its low expression levels in these tumors. Therefore, the development of more potent SIRT4 inhibitors as tools for functional research, and the potential use of SIRT4 activators for cancer therapy, may represent future directions in the field of small-molecule modulators targeting SIRT4.

SIRT5 is a mitochondrial enzyme with robust depropionylase and desuccinylase activities. Notably, SIRT5's preference for hydrolyzing the propionyl and succinyl groups from lysine residues in substrates is unique among the seven human SIRTs, guiding the design of specific SIRT5 regulatory modulators. Leveraging SIRT5's unique acyl preference, He *et al.* synthesized thiosuccinyl peptides and validated that the H3K9TSu peptide is a selective competitive inhibitor targeting SIRT5, marking the first specific inhibitor for SIRT5 32. Another study identified a series of peptide-based SIRT5 inhibitors, which are modified lysine side-chain derivatives of carbamoyl phosphate synthetase 1 (CPS1), acting on the NAD+ binding pocket and exerting specificity through SIRT5's acyl preference 117. Additionally, the co-crystal structures of these CPS1-derived peptides with SIRT5 were reported, elucidating their interaction patterns 117. The cell permeability of these peptide-based SIRT5 inhibitors is limited, constraining their use as selective chemical tools for studying SIRT5's biological functions in various diseases, particularly cancer.

MC3482, a selective SIRT5 inhibitor discovered in 2015, was employed to investigate the regulation of glutamine metabolism by SIRT5 in the breast cancer cell line MDA-MB-231 95. It has been found that MC3428-mediated SIRT5 inhibition led to an increase in intracellular ammonia, thereby promoting ammonia-induced autophagy and mitophagy 95. Given autophagy's dual role in tumor development, the impact of SIRT5 inhibition on breast cancer or other cancers requires further investigation. Moreover, a series of 3-arylthiosuccinylated and 3-benzylthiosuccinylated peptide derivatives were synthesized and conducted activity screening. Based on the analysis of the SIRT5/47 co-crystal structure, structural modifications yielded the biotinylated peptide 39.2 with potent inhibitory activity and selectivity 118. Interestingly, SIRT5 has been recently found to be significantly downregulated in pancreatic ductal adenocarcinoma (PDAC) and closely associated with PDAC progression and poor patient survival outcomes. Notably, the small molecule SIRT5 activator MC3183 exerts anti-PDAC effects through the SIRT5-GOT1-glutamine metabolism pathway, demonstrating promising antitumor activity both in vitro and in vivo, suggesting that developing more potent and specific small molecule activators to activate SIRT5 may be a promising new strategy for targeted PDAC therapy 92. Meanwhile, SIRT5, acting as an oncogene, promotes the onset and progression of acute myeloid leukemia (AML). Downregulation of SIRT5 or inhibition of SIRT5 activity using NRD167 induces apoptosis in AML cells through oxidative stress and the glutamine metabolism pathway, thereby restraining AML progression 119. 3-thioureido-propionic acid derivatives have been identified as novel inhibitors of SIRT5, exhibiting inhibitory activity through a mechanism distinct from conventional inhibitors that compete with acyl-lysine substrates 120. Thermal shift assays revealed that these derivatives, specifically compounds 36, 45, and 46, significantly enhance the thermal stability of SIRT5 without markedly affecting SIRT1-3 and SIRT6. This indicates the subtype selectivity of 3-thioureido-propionic acid derivatives for SIRT5 120. Additionally, balsalazide, an FDA-approved drug for treating inflammatory bowel disease, has also been found to effectively inhibit SIRT5 activity. However, due to its poor solubility, low oral bioavailability, and susceptibility to degradation, balsalazide is not considered a viable candidate for a targeted SIRT5 small molecule drug. Consequently, Glas *et al.* have performed structural modifications on the core azo moiety of balsalazide based on structure-activity relationship analysis, aiming to enhance the inhibitory activity against SIRT5 while retaining selectivity, resulting in two potential SIRT5 inhibitors, CG-220 and CG-232 121. Among the SIRT5 modulators for tumor therapy mentioned above, MC3183 is the only small-molecule activator specifically targeting SIRT5 (Fig.3). Additionally, several selective SIRT5 inhibitors (such as compounds 36, CG-220, CG-232, etc.) have not been further investigated for their anti-tumor activity and action mechanisms. While this section summarizes the small-molecule modulators of SIRT4 and SIRT5, the development of SIRT4 and SIRT5 modulators is still in its infancy. Currently, only SIRT5 modulators have been reported for potential cancer therapy, and none have progressed to clinical trials.

## 5. Concluding remarks and future directions

Mitochondrial SIRTs (SIRT3, SIRT4 and SIRT5) have been widely reported to regulate a variety of key biological processes, including gene expression, DNA damage repair, metabolism and survival, and have the Janus role in tumorigenesis, either tumor suppressive or oncogenic functions 122-124. In the context of cancer, mitochondrial SIRTs are involved in the metabolism, autophagy, invasion and metastasis of different types of cancer cells via their upstream and downstream key signaling pathways. Recently, a series of small-molecule compounds with the ability to regulate mitochondrial SIRTs have been considered to have the therapeutic potential on human cancers 125.

SIRT3 is an important mitochondrial deacetylation protein, which plays a key role in regulating protein acetylation level, maintaining mitochondrial integrity and energy metabolism. Highly acetylated modifications occur frequently in tumors, which contribute to the survival of most types of tumors. SIRT3 regulates tumor progression by adjusting the acetylated modifications to a normal state. In addition, SIRT3 has the ability of metabolic reprogramming, which also plays an important role in the occurrence and development of tumors. However, SIRT3 has been regarded as a double-edged sword for cancer therapy, which may increase the difficulty and risk of SIRT3 as a potential therapeutic target to some extent. Moreover, SIRT4 is involved in biological processes such as cell energy metabolism, genome integrity and carcinogenesis, and shows tumor inhibitory effect by inhibiting glutamine metabolism 126. The tumor suppressive function of SIRT4 makes people realize that tumor suppressor proteins can exist not only in nucleus and cytoplasm, but in mitochondria 127. It is believed that with the continuous progress of scientific research, SIRT4 will become a new target and direction in the field of tumor therapy. Additionally, SIRT5 can be involved in the regulation of substance metabolism, apoptosis, inflammatory reaction and other life activities through deacetylation, desucrylation, desmalonylation and deglutaranylation activities, and is closely related to the occurrence and development of cancer. At this stage, there are still many problems to be solved in the research of SIRT5. SIRT5 has a strong activity of de malonylation and de glutarylation, but its research is still in its infancy. The existing evidence shows that SIRT4 and SIRT5 have different levels of expression differences in different diseases, and whether these differences are the key factors causing cell physiological changes and affecting the prognosis of the disease is still an open question. At present, studies have proved that several key cellular metabolic regulatory pathways are affected by the expression of SIRT4 and SIRT5, but the specific mechanism of their participation is not completely clear, and as mitochondrial SIRT protein, the interaction and influence between them are not clear.

Accordingly, we have summarized about 21 small-molecule compounds targeting SIRT3 and 3 small-molecule compounds targeting SIRT5 for the current cancer therapy. Furthermore, the above-mentioned small-molecule inhibitors or activators of mitochondrial SIRTs have their different anti-cancer effects on distinctive cancer cell types, suggesting that the molecular mechanisms of mitochondrial SIRTs in cancer are complex and diverse, and their specific molecular mechanisms should need to be further explored. Due to the important role of SIRT4 and SIRT5 in metabolic regulation, they have the potential to become a new direction in the treatment of metabolic diseases and tumors, and the development of related agonists or inhibitors will also become a hot field. Advancing the understanding of mitochondrial SIRT molecular mechanisms and interactions is imperative for effectively leveraging their therapeutic potential. Targeting these proteins with small molecules holds promise as a precision medicine strategy, necessitating consideration of their interplay within the tumor microenvironment, the intricacies of molecular mechanisms, and their variable roles across different tumors or subtypes.

Currently, the development of small molecule anticancer drugs faces certain limitations, prompting recommendations for future drug development targeting mitochondrial SIRTs. Firstly, there should be a concerted effort to enhance drug selectivity and specificity to minimize normal cell damage and reduce the incidence of adverse reactions. Secondly, given the multifaceted roles of mitochondrial SIRTs in tumors and the challenge of tumor resistance, alongside the development of novel small molecule drugs targeting mitochondrial SIRTs, combination therapies with existing anticancer drugs could be explored to delay or overcome tumor drug resistance. Lastly, exploring the synergistic effects of combining small molecule drugs targeting mitochondrial SIRTs with immunotherapy represents a promising avenue for enhancing therapeutic outcomes, ultimately improving patient survival and quality of life.

In a nutshell, mitochondrial SIRTs have shown a clinical application prospect in cancer therapy, it is still necessary to clarify the biological functions of each member of the mitochondrial SIRT family through in-depth investigations from molecular mechanisms to targeted therapeutics. A better understanding of the structures and functions of mitochondrial SIRTs should be urgent. Based upon their structures and functions, a functional new therapeutic strategy would promote the further development of cancer drug research and development, so as to lay a solid foundation for new potential small-molecule drugs targeting mitochondrial SIRTs in the future.

## Figures and Tables

**Table 1:** Small-molecule compounds targeting SIRT3 for cancer therapy.

Name	Chemical Structure	Mechanism	Cancer cell type	EC_50_/ IC_50_	Pharmacological interventions	Preclinical tumor model	Ref.
Diethyl 1-(3,4-dimethoxybenzoyl)-4-phenyl-1,4-dihydropyridine-3,5-dicarboxylate	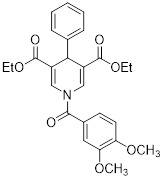	Increased the activity of glutamate dehydrogenase (GDH), the level of deacetylating K68- and K122-acMnSOD and reduced hypoxia and EMT targey modulation	Triple-negative breast cancer (TNBC), MDA-MB-231, thyroid cancer, CAL-62	/	MDA-MB-231 and CAL-62 are treated with 50 μM for 48 h.	/	102
Diethyl 1-(3,5-dimethoxybenzoyl)-4-phenyl-1,4-dihydropyridine-3,5-dicarboxylate	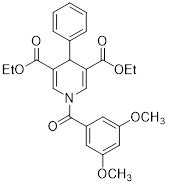	Increased the GDH activity, and reduced hypoxia and EMT targey modulation	TNBC, MDA-MB-231, thyroid cancer, CAL-62	IC_50_ = 3.64 μM IC_50_ = 16.7μM	MDA-MB-231 and CAL-62 are treated with 50 μM for 48 h.	/	102
ADTL-SA1215		Increased deacetylating SKP, activate PINK1-Parkin pathway and reversed Warburg effect	TNBC, MDA-MB-231	EC_50_ = 0.21 μM IC_50_ = 2.19 ± 0.16 μM	MDA-MB-231 is treated with 5 μM for 24 h. In MDA-MB-231 TNBC xenograft model with 25 mg/kg for 16 days.	/	103
MY-13	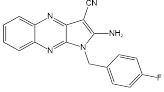	Regulated the SIRT3/Hsp90/AKT signaling pathway to induce autophagy-dependent cell death and apoptosis	Colorectal cancer, RKO, HCT116	IC_50_ = 5.32 μM IC_50_ = 14.71 μM	RKO is treated with 5.23 μM for 24 h. HCT116 is treated with 14.71μM for 24 h. In xenograft model with 40 and 50 mg/kg	Colorectal cancer	113
Honokiol	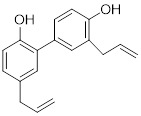	Reduced Kla level on CCNE2	Hepatocellular carcinoma, Huh7, TNBC, MDA-MB-231,	EC_50_ = 0.17 μM IC_50_ = 44.89 ± 1.86 μM	Huh7 is treated with 10 μM for 24 h. HCC mice is treated with 10 mg/kg.	Lung cancer	69
Compound 31	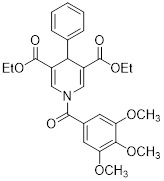	Increased the activity of GDH and the level of deacetylating K68- and K122-acMnSOD	TNBC, MDA-MB-231	/	MDA-MB-231 is treated with 50 μM for 24 h.	/	101
Resveratrol	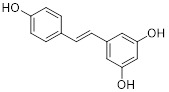	Inhibited SIRT3/AMPK/autophagy pathway	TNBC, MDA-MB-231	EC_50_ = 55.19 μM IC_50_ = 98.84 ± 2.16 μM	4T1 is treated with 25μM for 24 h. In MDA-MB-231 TNBC xenograft model with 40 mg/kg.	/	114
Melatonin	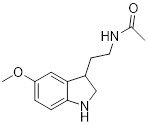	Activated pyruvate dehydrogenase complex and reversed Warburg effect	Lung cancer, A549, LLC, PC9,	IC_50_ = 2.5 mM, IC_50_ = 1.2 mM IC_50_ = 2.8 mM	PC9 and LCC is treated with 1.5 mM for 24 h. Lewis mouse model is treated with 10 mg/kg.	/	115
3-triazolylpyridine	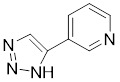	/	Leukemia, non-small cell lung cancer, colorectal cancer	IC_50_ = 16 nM	HeLa is treated with 4.7μM for 48 h. SK-MEL-28 is treated with 10 μM for 48 h.	Leukemia, ovarian, colon cancer	31
YC8-02	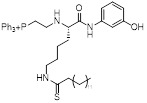	Increased mitochondrial protein acetylation and autophagy	Diffuse large B cell lymphomas	IC_50_ = 0.53 μM	DLBCL is treated with 10 μM for 24 h. C57BL/6 mice is treated with 30 mg/kg	/	70
JH-T4	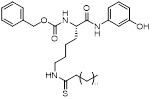	Increased mitochondrial protein acetylation and autophagy	Diffuse large B cell lymphomas	IC_50_ = 2.5 μM	DLBCL is treated with 10 μM for 24 h.	/	70
Biotin-TM3	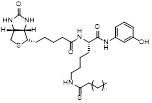	Increased mitochondrial protein acetylation and autophagy	Diffuse large B cell lymphomas	IC_50_ = 15 μM	DLBCL is treated with 10 μM for 24 h.	/	70
Compound 8a	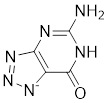	/	/	IC_50_ = 51.6 ± 1.28	/	/	104
Compound 15	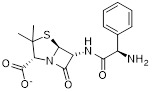	/	/	IC_50_ = 23.4 ± 2.11	/	/	104
4'-Bromo-resveratrol	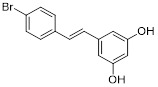	Induced apoptosis and a G0/G1 phase arrest through a metabolic reprogramming	Melanoma G361, SK-MEL-28, SK-MEL-2	IC_50_ = 143.0 ± 3.6 μM	G361, SK-MEL-28 and SK-MEL-2 are treated with 0.025 mM for 24 h.	/	107
Compound 15	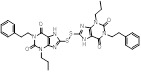	/	/	IC_50_ = 0.37 ± 0.05 μM	/	/	108
Butyrate	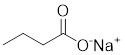	Induce apoptosis through activating PDHA1 hyperacetylation and reversing the Warburg effect	Colorectal cancer, HCT116	/	HeLa and HCT116 are treated with 5 mM for 24 h.	/	116
Compound P6	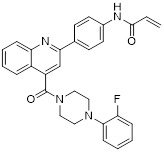	Induced a G0/G1 phase arrest	Mixed-lineage leukemias THP-1, MOLM-13, SEM and MV4-11	IC_50_ = 7.2 ± 0.5 μM	THP-1, MOLM-13, SEM and MV4-11 are treated with 0.9 μM for 24 h.	/	105
Compound P19	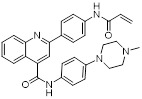	/	Mixed-lineage leukemias	/	THP-1, MOLM-13, SEM and MV4-11 are treated with 0.9 μM for 24 h.	/	105
Compound 8b	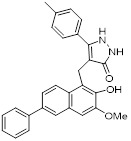	/	Diffuse large B-cell lymphoma, colon, HCT116, breast MCF7, nonsmall cell lung carcinoma NCI-H460	IC_50_ = 6 μM	NCI-H460 is treated with 1 μM for 24 h.	/	106
Kaempferol Apigenin	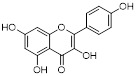	/	TNBC, T47D, MCF-7, MDA-MB-231, MDA-MB-468	IC_50_ = 123 ± 0.4 μg/mL IC_50_ = 132 ± 0.23 μg/mL IC_50_ = 24.85 ± 0.12 μg/mL IC_50_ = 27.6 ± 1.34 μg/mL IC_50_ = 25.01 ± 0.11 μg/mL	T47D, MCF-7, MDA-MB-231, MDA-MB-468 are treated with 20 μM for 24 h.	/	109

**Table 2:** Small-molecule compounds targeting SIRT5 for cancer therapy

Name	Chemical structure	mechanism	Cancer cell type	IC_50_	Pharmacological interventions	Preclinical tumor model	Ref.
MC3482	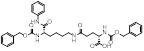	MC3428-mediated SIRT5 inhibition led to an increase in intracellular ammonia and promoted ammonia-induced autophagy and mitosis	MDA-MB-231	42% inhibition of SIRT5 at 50 μM	50μM MC3482 is treated in MDA-MB-231 for 24 h. No in vivo experiment information is available.	/	95
MC3183	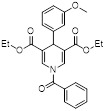	SIRT5-GOT1-glutamine metabolism pathway	CFPAC, FG, PANC1, S2-013, Canpan1, S2-007, T3M4, Patu8902, MIAPaCa-2, Capan2	IC_50_ = 25.4 - 236.9 μM	PDAC cells are treated with 10μM MC3183 for 24 h. The maximum concentration and half-life of MC3138 in plasma are approximately 230 μM and 5.059 hours, respectively. In the PDX tumor model of NOD-SCID mice, when the concentration of MC3183 in tumor tissue is around 100~200μM, the combined use of MC3183 and gemcitabine can effectively inhibit PDAC growth.	PDX models of pancreatic cancer	92
NRD167	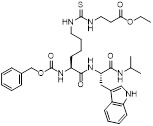	NRD167 induced apoptosis through oxidative stress and the glutamine metabolism pathway	SKM-1 and OCI-AML2	IC_50_ = 5-8 μM	5-10μM in AML cells and no in vivo experiment information is available.	Xenograft mouse models using CMK and OCI-AML3 cells	119

## Abbreviations

ACO2: aconitase 2
AMPK: 5'-adenosine monophosphate-activated protein kinase
ATTEC: autophagosome-tethering compound
AUTAC: autophagy-targeting chimera
CCNE2: cyclin E2
CPS1: carbamoyl phosphate synthetase 1
CRC: colorectal cancer
DLAT: dihydrolipoyllysine acetyltransferase
DLBCL: Diffuse large B cell lymphomas
DOX: doxorubicin
ECHS1: enoyl-coenzyme A (CoA) hydratase-1
EMT: epithelial-mesenchymal transitio
ETC: electron transport chai
FAO: fatty acid oxidation
GDH: Glutamate dehydrogenase
GLDC: glycine decarboxylase
GLS: glutaminase
GOT2: oxaloacetate transaminase 2
GSK-3β: glycogen synthase kinase-3β
HCC: hepatocellular carcinoma
HIF1α: hypoxia-inducible factor-1α
IDH2: isocitrate dehydrogenase 2
LONP1: Lon protease-1
MnSOD: manganese superoxide dismutase
MPP: mitochondrial processing peptidase
NAD+: nicotinamide adenine dinucleotide
NSCLC: non-small cell lung cancer
OXPHOS: oxidative phosphorylation
PDH: pyruvate dehydrogenase
PHD: prolyl hydroxylase
PPP: pentose phosphate pathway
PROTAC: proteolysis targeting chimera
PYCR1: pyrroline-5-carboxylate reductase 1
R5P: ribose-5-phosphate
RCD: regulated cell death
RIF1: replication timing regulatory factor 1
ROS: reactive oxygen species
SHMT2: serine hydroxymethyltransferase 2
SIRT3: Sirtuin 3
SIRT4: Sirtuin 4
SIRT5: Sirtuin 5
TCA: tricarboxylic acid
α-KG: α-ketoglutarate
